# Mechanistic Insights into Ameliorating Effect of Geraniol on d-Galactose Induced Memory Impairment in Rats

**DOI:** 10.1007/s11064-022-03559-3

**Published:** 2022-03-02

**Authors:** Marwa Mohamed Atef, Marwa Nagy Emam, Rehab E. Abo El Gheit, Eman M. Elbeltagi, H. A. Alshenawy, Doaa A. Radwan, Reham L. Younis, Rania Nagi Abd-Ellatif

**Affiliations:** 1 Medical Biochemistry Department, Faculty of Medicine, Tanta University, El Geesh Street, Tanta, 31511 Egypt; 2 Physiology Department, Faculty of Medicine, Tanta University, Tanta, Egypt; 3 Histology Department, Tanta University, Tanta, Egypt; 4 Pathology Department, Faculty of Medicine, Tanta University, Tanta, Egypt; 5 Anatomy and Embryology Department, Faculty of Medicine, Tanta University, Tanta, Egypt

**Keywords:** Geraniol, d-Galactose, Memory impairment, ER stress, Oxidative stress

## Abstract

Geraniol (GE), an important ingredient in several essential oils, displayed pleiotropic biological activities through targeting multiple signaling cascades. In the current study, we aimed to examine the protective effect of GE on d-galactose (d-gal) induced cognitive impairment and explore the underlying mechanisms. Forty male Wistar rats (8 weeks old) were randomly categorized into 4 groups; Group I (saline + vehicle [edible oil]), group II (saline + geraniol) (100 mg/kg/day orally), group III (d-galactose) (100 mg/kg/day subcutaneously injected), and group IV (d-galactose + geraniol). Behavioral impairments were evaluated. Brain levels of malondialdehyde (MDA) and reduced glutathione (GSH) as well as superoxide dismutase (SOD) and acetylcholinesterase (AchE) activities were estimated. The levels of inflammatory markers [tumor necrosis factor-alpha (TNF-α), interleukin (IL)-1β, IL-6, and nuclear factor kappa beta (NF-kβ)], endoplasmic reticulum stress sensors [inositol requiring protein 1(IRE1) and protein kinase RNA–like endoplasmic reticulum kinase (PERK)], brain-derived neurotrophic factor (BDNF), and mitogen-activated protein kinases (MAPK) pathway were measured by ELISA. Also, hippocampal histopathological assessment and immunohistochemical analysis of glial fibrillary acidic protein (GFAP) and caspase-3 were performed. Glucose regulated protein 78 (GRP78) and C/EBP homologous protein (CHOP) mRNA expression and protein levels were assessed. GE effectively ameliorated aging-related memory impairment through increasing GSH, BDNF, Ach levels, and SOD activity. Additionally, GE treatment caused a decrease in the levels of MDA, inflammatory mediators, and ER stress sensors as well as the AchE activity together with concomitant down-regulation of GRP78 and CHOP mRNA expression. Moreover, GE improved neuronal architecture and rat's spatial memory; this is evidenced by the shortened escape latency and increased platform crossing number. Therefore, GE offers a unique pharmacological approach for aging-associated neurodegenerative disorders.

**Graphical Abstract:**

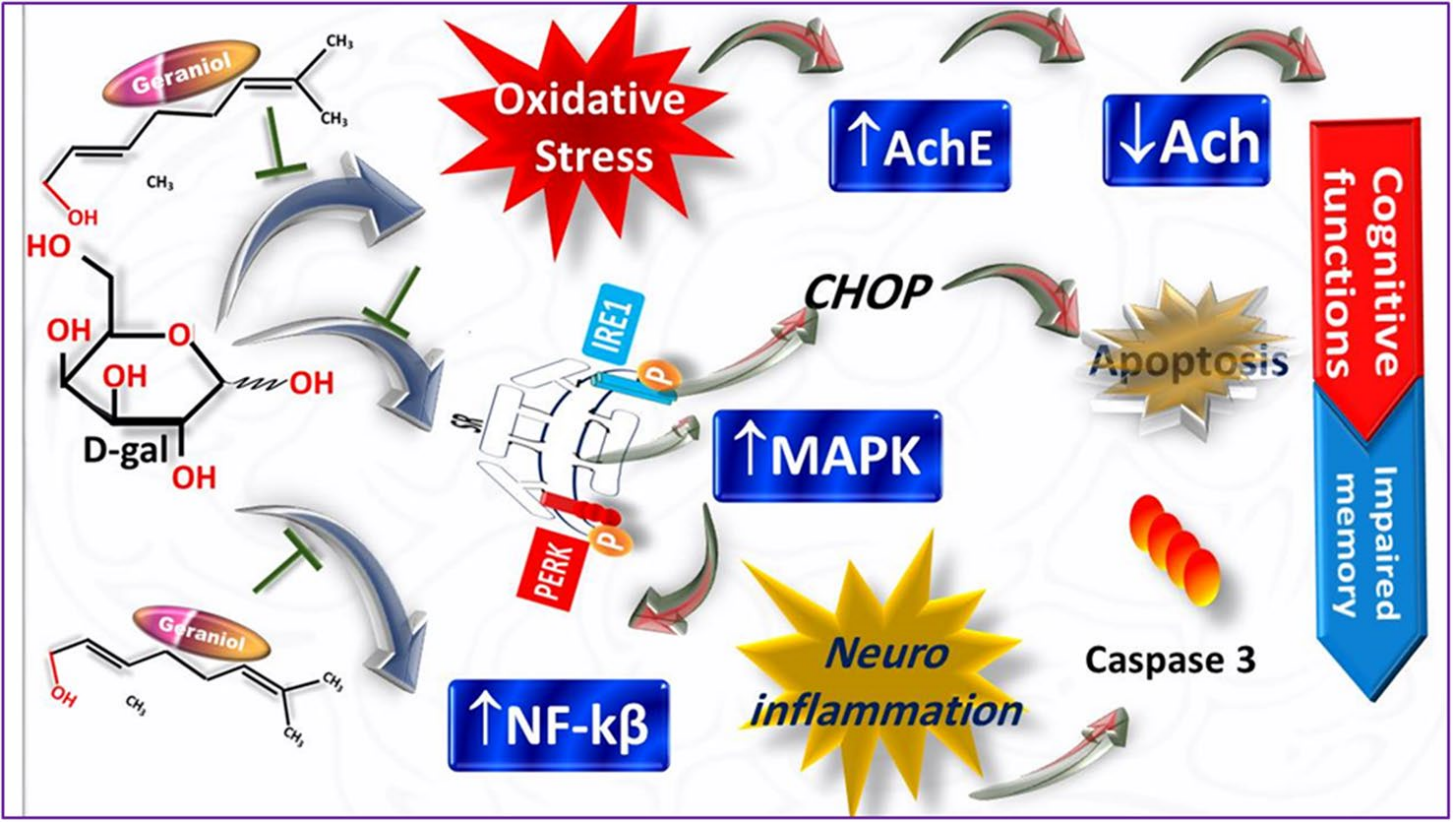

## Introduction

It is predicted that by the year 2025, the old population (over 65 years) will be more than 800 million. The increasing number of old people will lead to an increase in disability and illness [1]. Thus, studying pathophysiological mechanisms of aging and associated diseases is an important challenge for medical gerontology [2].

Aging is a complex multidimensional phenomenon causing progressive molecular and physiological dysfunctions [1]. Aging is accompanied by physiological insults as homeostatic imbalance and several pathological disorders such as anxiety, depression-like behavior, and memory impairment [3].

While aging has numerous theories, there is growing evidence that the notion of free radical damage is the most accepted mechanism of aging. This theory states that the buildup of free radicals or reactive oxygen species (ROS), due to oxidative stress, is the leading cause of tissue and cellular damage associated with alterations in genetic apparatus, causing cell death and aging. ROS-induced cellular damage takes place as a result of the interaction of free radicals with DNA, proteins, and lipids, causing plentiful deleterious effects such as enzymatic destruction, inflammatory damage, cellular apoptosis, and finally cell senescence [4].

The endoplasmic reticulum (ER) is a major cellular organelle responsible for the synthesis and folding of around one-third of the proteome. Many endogenous and exogenous environmental conditions can disrupt ER homeostasis, causing accumulation of unfolded/misfolded proteins which leads ultimately to ER stress [5]. Unfolded/misfolded proteins have been implicated to play a pivotal role in normal aging and aging-associated cognitive dysfunction. ER stress activates unfolded protein response (UPR) to restore ER homeostasis, preventing further cellular damage. However, the failure of ER homeostasis causes the UPR to trigger apoptosis in such cells. Thus, targeting ER stress-induced apoptosis conveys an important therapeutic approach in many disorders [6].

d-Galactose, a naturally occurring monosaccharide (aldohexose), is typically present in the body fluid. The metabolism of d-galactose depends on its concentration. In high concentration, it is metabolized by d-galactose oxidase to aldose and hydrogen peroxide. These substances act as a potent catalyst for the production of oxygen-derived free radicals [7]. Copious studies revealed that chronically administrated d-galactose for 6–10 weeks caused a state of oxidative stress, giving manifestations which mimic natural aging such as mitochondrial dysfunction, loss of proteins, hippocampal neuronal damage, and cognitive impairment in rodents [8]. Therefore, d-galactose-induced aging is the commonly used model for investigating brain aging and anti-aging therapeutic agents [3].

Although aging has been recognized as an inevitable process, several studies provide evidence that the aging process can be modified. This modulation can be achieved by genetic or pharmacological interventions of certain genes or signaling pathways involved in the process of aging [9].

Numerous studies explored the protective antioxidant effects of some natural plants against oxidative stress involved in the pathophysiology of some impairment like aging. One of these antioxidants is geraniol (GE) (3, 7-dimethyl-2, 6-octadien-1-ol). GE has been identified as an acyclic monoterpene alcohol which is the main ingredient of palmrosa, lavender, rose, ginger, lemon, and orange essential oils. This antioxidant has been reported to have an array of biological and pharmacological effects as anti-microbial, anti-ulcer, anti-inflammatory, anti-oxidant, and neuroprotective effects [10]. To the best of our knowledge, the effect of GE on d-gal-induced memory impairment has not been yet investigated. Therefore, our study was designed to investigate the protective effect of GE against d-gal induced memory impairment and try to elucidate the underlying mechanisms.

## Materials and Methods

### Chemicals

d-Galactose, geraniol, and most of the chemicals used in the present study were purchased from Sigma-Aldrich Chemicals (St. Louis, MO, USA).

### Animals

Fifty male Wistar rats (8 weeks old) (weight 200–240 g) were purchased from the animal breeding laboratory, Faculty of Science, Tanta University, Egypt. Rats were kept in wire mesh fully ventilated cages (3 rats per cage) at a suitable temperature (25 ± 2 °C) and 50–60% relative humidity with alternative day/light cycles of 12 h for each. The animals had free access to chow and water ad libitum throughout the experimental period. Rats were acclimatized for these conditions for one week before the experiment. The present experiment was conducted in accordance with the National Institutes of Health Guide for the Care and Use of Laboratory Animals and the Ethical Animal Research Committee of Tanta University (approval No: 34098/9/20).

### Experimental Design

One week following the initial acclimatization, all animals were screened by Morris water maze test to select the qualified rats that succeeded to search the hidden platform within the 90-s limit [11]. The rats failed to find the hidden platform within 90 s were excluded from the experiment. Forty qualified rats were allocated randomly into 4 groups (10 rats per each group).

Group I (Control group): The rats received a subcutaneous injection of 0.9% saline (vehicle) at a dose of 0.2 ml/day and equal volume of edible oil orally for 8 weeks. Group II (geraniol group): The rats received a subcutaneous injection of 0.9% saline (vehicle) at a dose of 0.2 ml/day and geraniol (100 mg/kg/day) dissolved in edible oil orally for 8 weeks [12]. Group III (d-galactose group): The rats were subcutaneously injected with d-gal (100 mg/kg/day) for 8 consecutive weeks [13]. Group IV (d-galactose + geraniol group): The rats received a subcutaneous injection of d-gal in the same dose as group III along with geraniol (100 mg/kg/day) dissolved in edible oil orally for 8 weeks [12]. The decided dose of GE was selected upon a preliminary pilot study.

### Behavioural Tests

**Fig. 1:**
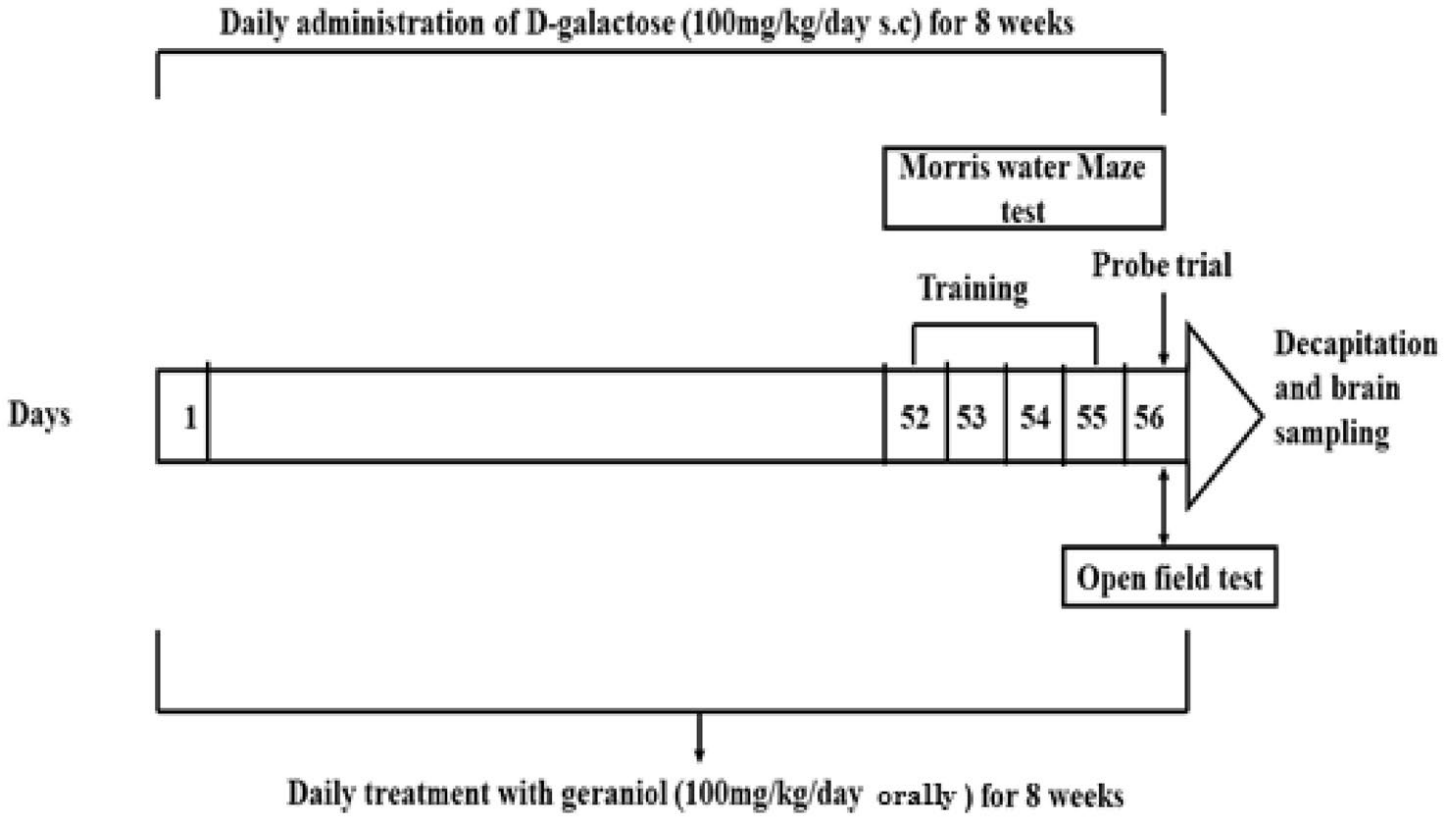
Simple schematic representation of the experimental protocol

All behavioural tests were done in the light phase of the day (between 8 am and 3 pm). Rats were subjected to Morris water maze (MWM) test 5 days before the end of our experiment (on day 52). MWM training was carried out on 4 consecutive days (days 52–55). On the 5th day (day 56), the probe trial was done. Open field test was conducted after the last MWM test (on day 56) to test locomotor activity (Fig. 1).

#### Morris Water Maze Test

Morris water maze test was performed to examine spatial learning and memory abilities of the studied rats. The maze consisted of a large circular pool (100 cm diameter and 40 cm in height) and was filled with water (25 ± 1 °C) to 30 cm depth. This pool was divided virtually into 4 equal quadrants and a circular platform (9 cm in diameter) was hidden 2 cm below the water surface at the center of a specific quadrant. In the acquisition phase, rats were subjected to 2 training trials per day for 4 successive days. During each trial, rats were allowed to find the hidden platform in the target quadrant. The rats succeeded to find the platform within 90 s were allowed to stay in it for 10 s. If any of the rats failed to find the platform within the allocated time, it was manually guided to the platform and placed in it for 10 s [14]. Escape latency (EL) was measured as the time taken by the animals to reach the hidden platform. Twenty four hours after the last training trial (day 56), probe test (retrieval test) was carried out where the platform was removed and each rat was put in the pool (in a quadrant just opposite to the platform quadrant) for 90 s. Frequencies of passing through the former position of the platform were tested in 90 s, expressed as the number of platform crossing, and used as an index of retrieval.

#### Open Field Test

This test was used to assess the locomotor activity and exploratory behavior of rats. Open field (81 × 81 × 28 cm) was constructed; its floor was divided into 25 squares by black lines. Rats were put in the center of the open field to adapt the apparatus for 1 min. Then, the locomotor activity was examined by recording the number of squares crossed and the total number of rearing done in 5 minutes [15].

### Tissue Sampling

Twenty-four hours following the completion of behavioral tests, all animals were anesthetized with sodium pentobarbital (60 mg/kg., *i.p*) and sacrificed by cervical decapitation. The animals’ skull vaults were dissected out and temporal lobes were sagittally divided into left and right hemispheres. In each group, the left half of the brain was fixed in 10% neutral buffered formalin for 24 h for histological examination. The right half was thoroughly washed with 0.9% saline and stored at – 80 °C till the tissue homogenate and nuclear extract were prepared.

### Preparation of Brain Homogenate

Brains were homogenized in 1/5 (w/v) phosphate-buffered saline (PBS) in an ice bath to collect the homogenate. This homogenate was centrifuged (8000×*g* for 10 min at 4 °C) and the supernatant aliquot was kept for further biochemical analysis.

### Preparation of Nuclear Extracts

Using Membrane, Nuclear and Cytoplasmic Protein Extraction Kits obtained from Bio Basic INC., Canada) following the kit protocol.

### Biochemical Analysis

#### Assay of Cholinergic Function

Acetylcholine (Ach) level in brain homogenate as well as AchE activity were estimated by using commercially available kits (Nanjing Jiancheng Bioengineering Research Institute, Nanjing, China) according to the manufacturers' instructions.

#### Assay of Oxidative Stress and Antioxidant Defense

Tissue MDA level was tested by the spectrophotometric method depending on the calculation of MDA concentration using an extinction coefficient of MDA–TBA complex (1.56 × 105 M^−1^ cm^−1^) [16]. Briefly, 250 μl of brain tissue homogenate was shaken with 1.25 ml of TCA (20%) in a 10 ml centrifuge tube. Then, 0.67% of TBA (0.5 ml) was added, shaken, and heated in a boiling water bath for 30 min followed by rapid cooling. Next, 2.0 ml of N-butanol was added and the tubes were shaken and centrifuged at 3000 rpm for 10 min. The resultant N-butanol layer was separated and absorbance was determined at 532 nm against a blank using a semiautomatic BTS-350 Biosystems spectrophotometer.

Tissue SOD activity was examined by the spectrophotometric method based on the previously described technique [17]. Reduced glutathione (GSH) level in brain tissue homogenate was measured colorimetrically according to the previously described method [18]. This method is based on the reduction of 5, 5′ dithiobis 2-nitrobenzoic acid (DTNB) by GSH to produce a yellow compound which is directly proportional to GSH concentration and absorbance was measured at 405 nm.

#### Assay of Inflammatory Cytokines

TNF-α, IL-1β, and IL-6 were estimated using commercially available ELISA kits purchased from (RayBiotech Inc., GA, USA) based on the manufacturers' instructions.

#### Assay of Nuclear Factor-Kappa B (NF-kβ) and Mitogen-Activated Protein Kinase (MAPK) Signaling Pathway in Brain Homogenate [19]

Brain homogenate was subjected to commercial kit for immunosorbent assay (ELISA) for the detection of protein levels of NF-kβ (MyBioSource, San Diego, CA, USA) and phosphorylated (phospho) form of MAPK cascade, involving phosphorylated p38MAPK (at Thr^180^/Tyr^182^) and phospho-JNK (Thr^183^/Tyr^185^) (eBioscience, San Diego, CA, USA) according to the manufacturers' instructions.

#### Assessment of Brain-Derived Neurotrophic Factor (BDNF)

BDNF in the brain was measured using commercially available rat ELISA kits (MyBioSource, San Diego, CA, USA, cat.) according to the manufactures’ guidelines.

#### Assay of ER Stress Sensors Levels

Protein kinase RNA-like endoplasmic reticulum kinase (PERK), inositol requiring enzyme-1 (IRE1) and 78-kDa glucose-regulated protein (GRP78) levels were assayed by ELISA using the commercially available rat kits (MyBioSource, San Diego, CA, USA) according to the manufacturer’s guidelines. C/EBP homologous protein (CHOP) level was assessed in nuclear extracts using ELISA Kit that was obtained from (MyBioSource, San Diego, CA, USA) following kit protocol.

### Molecular Assessment

#### RNA Extraction, cDNA Synthesis, and Real Time PCR

Frozen hippocampal tissue was used to extract total RNA with the help of Gene JET RNA Purification Kit (#K0731, Thermo Fisher Scientific Inc., USA). Total RNA (5 μg) was reverse transcribed into cDNA using RevertAid H Minus Reverse Transcriptase Kit (Cat#K1632, Thermo Fisher Scientific Inc., USA) according to the manufactures’ protocol. With the guidance of manufacturers, PCR reactions were done using QuantiTect SYBR-Green PCR kit (Qiagen). Quantification of the target mRNA transcripts was accomplished in relation to the housekeeping gene β-actin (internal control). The sequences of specific primers were: rat GRP78 (Gene Bank Accession No. NM_013083.2): forward primer (5 GTTCTGCTTGATGTGTGTCC-3) and reverse primer (5’ TTTGGTCATTGGTGATGGTG-3′); rat CHOP: forward primer (5-GTA CCT ATG TTT CAC CTC CTG G-3) and reverse primer (5-TGG AAT CTG GAG AGT GAG GG-3); rat β- Actin (Gene Bank accession No.NM_031144.3): forward primer (5-GGCTGTGTTGTCCCTGTAT-3′) and reverse primer (5- CCGCTCATTGCCGATAGTG-3′). The relative gene expression was analyzed using the threshold cycle 2^−ΔΔCt^ method.

### Histological Assessment

The left half of each brain fixed in 10% neutral buffered formalin for 24 h was washed and dehydrated with an ascending grade of alcohol, cleared in xylol, then embedded in paraffin. 5 μm thick sections were prepared and stained with hematoxylin and eosin (H&E) according to Bancroft and Layton [21] for studying the histological structure of the CA1 region of hippocampus.

### Immunohistochemical Analysis

From hippocampal paraffin blocks, thick sections of 5 μm were deparaffinized in xylene. Rehydrated sections were incubated with 3% hydrogen peroxide in humidified boxes to block the activity of endogenous peroxidases. Microwave-assisted antigen retrieval was performed for 20 min. Some sections were incubated overnight at 4ºC in a humid room with polyclonal rabbit antibodies of glial fibrillary acidic protein (GFAP) (Catalog Number 16825-1-AP, Proteintech Group Inc.) and the other sections were incubated with caspase-3 antibodies (Catalog Number A71049, EpiGentek). Sections were washed and incubated for another 1 h in biotinylated goat anti-rabbit secondary antibodies diluted 1:500 in PBS and then further incubated with avidin–biotin-horseradish peroxidase complex for 15 min at 37 °C. The sections were then rinsed with PBS twice, incubated with 2 drops of diaminobenzidine tetrahydrochloride substrate chromogen solution (DAB) till the desired brown colour was obtained. Meyer's hematoxylin was used as a counterstaining for the background. The CA1 region of the hippocampus is examined in all of the studied groups. A positive reaction for caspase-3 appeared as a brown cytoplasmic accumulation in the granular cells where the positive reaction for GFAP was visualized as brownish cytoplasmic granules inside astrocytes. Negative controls were prepared using the same steps except that PBS was applied instead of primary antibodies [21].

### Morphometric Study

Immunohistochemical quantification was conducted by using an image analysis software (Image J, 1.46a, NIH, USA). Ten non-overlapping randomly selected fields from each slide were measured at a magnification of 400 for quantitative evaluation of the mean area percentage of GFAP and caspase-3 immunostaining reaction [calculated as the area of positive immunohistochemical reaction * 100/total area].

### Statistical Analysis

All results were expressed as mean ± standard deviation (SD). Data were analyzed using the statistical package for social sciences statistical analysis software (IBM SPSS Statistics for Windows, IBM Corp, and Version 23.0. Armonk, NY, USA). The analysis was performed using one-way analysis of variance (ANOVA) followed by Tukey's test. P value ≤ 0.05 was considered statistically significant.

## Results

### The Effect of GE Treatment on Behavioural Tests in All the Studied Groups

Memory and learning impairments induced by d-gal were assessed by Morris Water Maze test (MWM). During the acquisition phase, there was a significant prolongation in the mean escape latency to the hidden platform (20.82 ± 2.95) together with a significant decrease in the number of platform crossing in the probe trial (1.33 ± 0.44) in the d-gal group (P < 0.05) compared to control (12.61 ± 1.8) & (3.83 ± 0.56) and geraniol groups (12.19 ± 1.62) & (3.7 ± 0.54). However, the aforementioned results were reversed in the d-galactose + geraniol group (13.1 ± 2.44) & (3.45 ± 0.44) (Table 1). Open field test revealed that the total rearing (8.78 ± 1.44) and number of squares crossed in 5 min (37.33 ± 6.01) were significantly decreased in d-gal group (P < 0.05) compared to control (15.8 ± 2.72) & (62.55 ± 11.27) and geraniol groups (15.53 ± 2.7) & (61.8 ± 11.3), indicating impaired exploratory and ambulatory activities caused by chronic d-gal administration. GE treatment of d-gal group resulted in a significant increase in total rearing (14.95 ± 2.37) and number of squares crossed in 5 min (61 ± 8.86) (P < 0.05) compared to the d-gal group (Table 1).

**Table 1:** Effect of geraniol treatment on behavioural tests in all the studied groups

Groups/parameters	Morris water maze test		Open field test	
	Escape latency (s)	No. of platform crossing	Total rearing/5 min	Squares crossed in 5 min
Group I (control group)	12.61 ± 1.8	3.83 ± 0.56	15.8 ± 2.72	62.55 ± 11.27
Group II (geraniol group)	12.19 ± 1.62	3.7 ± 0.54	15.53 ± 2.7	61.8 ± 11.3
Group III (d-galactose group)	20.82 ± 2.95*^,#^	1.33 ± 0.44*^,#^	8.78 ± 1.44*^,#^	37.33 ± 6.01*^,#^
Group IV (d-galactose + geraniol group)	13.1 ± 2.44^&^	3.45 ± 0.44^&^	14.95 ± 2.37^&^	61 ± 8.86^&^

Data were expressed as mean ± standard deviation. (n = 10/group). Statistical analysis was carried out using one-way ANOVA with Tukey's post hoc test, SPSS computer program

*Mean significant difference vs control group (P < 0.05)

^#^Mean significant difference vs geraniol group (P < 0.05)

^&^Mean significant difference vs d-galactose group (P < 0.05)

### The Effect of GE Treatment on Cholinergic Functions in All the Studied Groups

Chronic d-gal administration caused impaired cholinergic function in aging rats as revealed by a significant increase in AchE activity (4.92 ± 0.46) together with a significant decrease in Ach level (4.5 ± 0.75) in the d-gal group compared to control (2.9 ± 0.23) & (6.2 ± 0.61) and geraniol (2.8 ± 0.22) & (6.04 ± 0.43) groups. On the other hand, GE administration with d-galactose resulted in the reversal of abnormalities in AchE activity and Ach level (3.04 ± 0.42 & 5.75 ± 0.75, respectively) (Table 2).

**Table 2:** Effect of geraniol treatment on brain redox status parameters, cholinergic functions, and inflammatory markers in all the studied groups

Parameters/groups	Group I (control group)	(Group II) (geraniol group)	(Group III) (d-galactose group)	(Group IV) (d-galactose + geraniol group)
Ach level (U/mg protein)	6.2 ± 0.61	6.04 ± 0.43	4.5 ± 0.75*^,#^	5.75 ± 0.75^&^
AchE activity (U/mg protein)	2.9 ± 0.23	2.8 ± 0.22	4.92 ± 0.46*^,#^	3.04 ± 0.42^&^
MDA level (nmol/mg protein)	1.5 ± 0.28	1.44 ± 0.29	4.8 ± 0.73*^,#^	1.75 ± 0.48^&^
SOD activity (U/mg protein)	0.88 ± 0.13	0.81 ± 0.12	0.68 ± 0.12*	0.86 ± 0.09^&^
GSH level (µmol/mg protein)	0.2 ± 0.058	0.19 ± 0.036	0.07 ± 0.028*^,#^	0.18 ± 0.055^&^
NF-kβ (ng/mg protein)	79.7 ± 6.5	79.54 ± 6.33	173.9 ± 16.5*^,#^	115.5 ± 11.25 *^,#,&^
TNF-α (pg/mg protein)	17.77 ± 2.55	15.77 ± 2.56	31.55 ± 4.17*^,#^	22.4 ± 4.62*^,#,&^
IL-1β (pg/mg protein)	44.1 ± 5.79	41.74 ± 5.55	31.55 ± 4.17*^,#^	102.15 ± 9.5*^,#,&^
IL-6 (pg/mg protein)	20.19 ± 2.9	19.2 ± 2.98	35.4 ± 3.63*^,#^	26.46 ± 3.38*^,#,&^

Data were expressed as mean ± standard deviation. (n = 10/group). Statistical analysis was carried out using one-way ANOVA with Tukey's post hoc test, SPSS computer program

*Ach level* acetylcholine level, *AchE activity* acetylcholinesterase enzyme activity, *MDA* malondialdehyde, *GSH* reduced glutathione, *NF-κβ* nuclear factor kappa beta, *SOD* superoxide dismutase, *TNF-α* tumor necrosis factor-alpha, *IL-1β* interleukin 1 beta, *IL-6* interleukin 6

*Mean significant difference vs control group (P < 0.05)

^#^Mean significant difference vs geraniol group (P < 0.05)

^&^Mean significant difference vs d-galactose group (P < 0.05)

### The Effect of GE Treatment on Brain Redox Status Parameters and Inflammatory Markers in All the Studied Groups

Redox standard markers were estimated to assess oxidative stress in the brain. As revealed in Table 2, chronic d-gal administration caused a state of oxidative stress in the brain that was detected by a significant increase in MDA level (4.8 ± 0.73) together with a significant decrease in SOD activity (0.68 ± 0.12) and GSH level (0.07 ± 0.028) (P < 0.05) compared to the control and geraniol groups. On the other hand, there was a significant decrease in MDA level (1.75 ± 0.48) associated with a significant increase in SOD activity (0.86 ± 0.09) and GSH level (0.18 ± 0.055) in the d-galactose + geraniol group compared to the d-gal group.

The levels of inflammatory mediators including NF-kβ, TNF-α, IL-1β, and IL-6 were estimated to confirm the role of chronic inflammation in neurodegenerative changes developed in the aging process. As shown in Table 2, the levels of these inflammatory mediators were increased significantly (P < 0.05) following chronic d-gal administration (173.9 ± 16.5, 31.55 ± 4.17, 31.55 ± 4.17 & 35.4 ± 3.63, respectively), while a significant decrease in these markers was observed in the d-galactose + geraniol group compared to the d-gal group. (115.5 ± 11.25, 22.4 ± 4.62, 102.15 ± 9.5 & 26.46 ± 3.38 respectively).

### The Effect of GE Treatment on Mitogen Activated Protein Kinase (MAPK) Signaling Pathway in Brain Homogenate

**Fig. 2:**
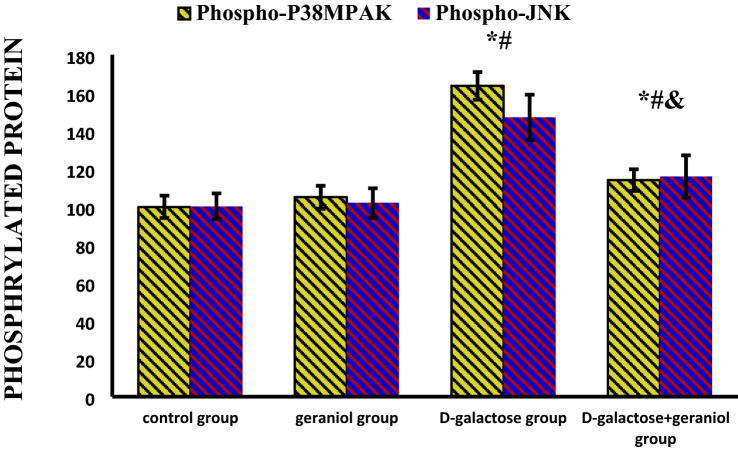
Effect of geraniol treatment on brain phosphorylated (phospho) mitogen-activated protein kinases (MAPKs, P^38MAPK^, and JNK) proteins in all the studied groups. Data were expressed as mean ± standard deviation. (n = 10/group). Statistical analysis was carried out using one-way ANOVA with Tukey's post hoc test, SPSS computer program. *Mean significant difference vs control group (*P* < 0.05). ^#^Mean significant difference vs geraniol group (*P* < 0.05). ^&^Mean significant difference vs d-galactose group (*P* < 0.05). *JNK* c-Jun N-terminal kinase.

Phosphorylated levels of P38MAPK and C-Jun N-terminal (JNK) kinases were significantly increased in the d-gal group (163.65 ± 7.26 & 147.02 ± 11.98, respectively) compared to the control and geraniol groups. On the other hand, GE treatment of d-gal group reversed this increase (113.94 ± 5.69 & 115.84 ± 11.14, respectively) (Fig. 2).

### The Effect of GE Treatment on Brain Derived Neurotrophic Factor (BDNF) in All the Studied Groups

Table (3) showed that there was a significant decrease in BDNF level in the d-gal group (5.61 ± 1.51**)** in comparison to the control (9.8 ± 1.51) and geraniol (9.54 ± 0.97) groups. In contrast, GE treatment of the d-gal group caused a significant increase in the BDNF level (7.8 ± 1.38**)**.

### The Effect of GE Treatment on ER Stress Sensors in All the Studied Groups

**Fig. 3:**
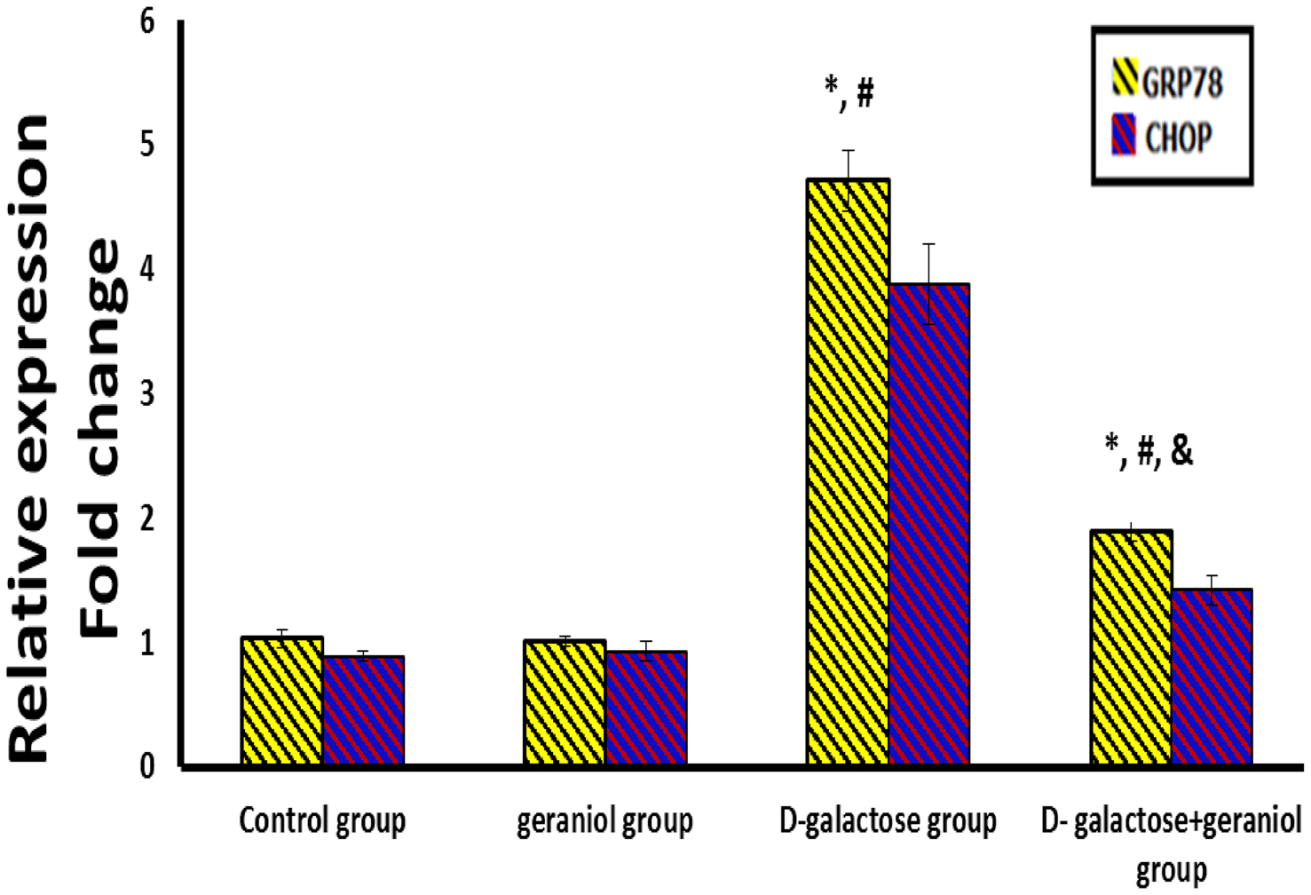
Effect of geraniol treatment on relative hippocampal *GRP78* and *CHOP* mRNA expression in all the studied groups. Data were expressed as mean ± standard deviation. (n = 10/group). Statistical analysis was carried out using one-way ANOVA with Tukey's post hoc test, SPSS computer program. *Mean significant difference vs control group (*P* < 0.05). ^#^Mean significant difference vs geraniol group (*P* < 0.05). ^&^Mean significant difference vs d-galactose group (*P* < 0.05). *GRP78* 78-kDa glucose-regulated protein, *CHOP* C/EBP homologous protein

Chronic d-gal administration resulted in a significant increase in GRP78 and CHOP mRNA expression (4.71 ± 0.25 & 3.88 ± 0.32, respectively) and protein levels (14.96 ± 1.75 & 4.09 ± 0.37 respectively) in comparison to the control and geraniol groups (P < 0.05), while GE treatment of d-gal group caused a significant decrease in the expression of these parameters (1.89 ± 0.08 & 1.42 ± 0.12, respectively) and protein levels (7.75 ± 1.91 & 2.39 ± 0.51 respectively) when compared to the d-gal group (Table 3 & Fig. 3).

**Table 3:** Effect of geraniol treatment on BDNF and ER stress sensors in all the studied groups

Parameters/groups	Group I (control group)	(Group II) (geraniol group)	(Group III) (d-galactose group)	(Group IV) (d-galactose + geraniol group)
BDNF level (pg/mg protein)	9.8 ± 1.51	9.54 ± 0.97	5.61 ± 1.51*^,#^	7.8 ± 1.38*^,#^^,&^
PERK level (pg/mg protein)	99.08 ± 8.27	99.76 ± 10.89	274.61 ± 12.01*^,#^	170.4 ± 16.22*^,#,&^
IRE1 level (pg/mg protein)	67.03 ± 8.08	66.91 ± 8.24	217.44 ± 15.27*^,#^	116.89 ± 13.07*^,#,&^
GRP78 level (ng/mg protein)	3.26 ± 0.81	3.28 ± 1.02	14.96 ± 1.75*^,#^	7.75 ± 1.91*^,#^^,&^
CHOP level (ng/mg protein)	1.32 ± 0.22	1.34 ± 0.25	4.09 ± 0.37*^,#^	2.39 ± 0.51*^,#^^,&^

Data were expressed as mean ± standard deviation. (n = 10/group). Statistical analysis was carried out using one-way ANOVA with Tukey's post hoc test, SPSS computer program

*BDNF* brain derived neurotrophic factor, *CHOP* C/EBP homologous protein, *GRP78* 78-kDa glucose-regulated protein, *PERK* protein kinase RNA-like endoplasmic reticulum kinase, *IRE1* inositol requiring enzyme-1

*Mean significant difference vs control group (*P* < 0.05)

^#^Mean significant difference vs geraniol group (*P* < 0.05)

^&^Mean significant difference vs d-galactose group (*P* < 0.05)

Also, inositol requiring protein 1 (IRE1) and protein kinase RNA-like endoplasmic reticulum kinase (PERK), the main ER transmembrane sensors, were significantly increased in the d-gal group (217.44 ± 15.27 & 274.61 ± 12.01, respectively) compared to the control and geraniol groups (P < 0.05). Meanwhile, the d-galactose + geraniol group showed a significant decrease in these sensors (116.89 ± 13.07 & 170.4 ± 16.22, respectively) compared to the d-gal group (Table 3).

### The Effect of GE Treatment on Hippocampal Histopathological Alteration in d-Gal-Induced Aging Rats

**Fig. 4:**
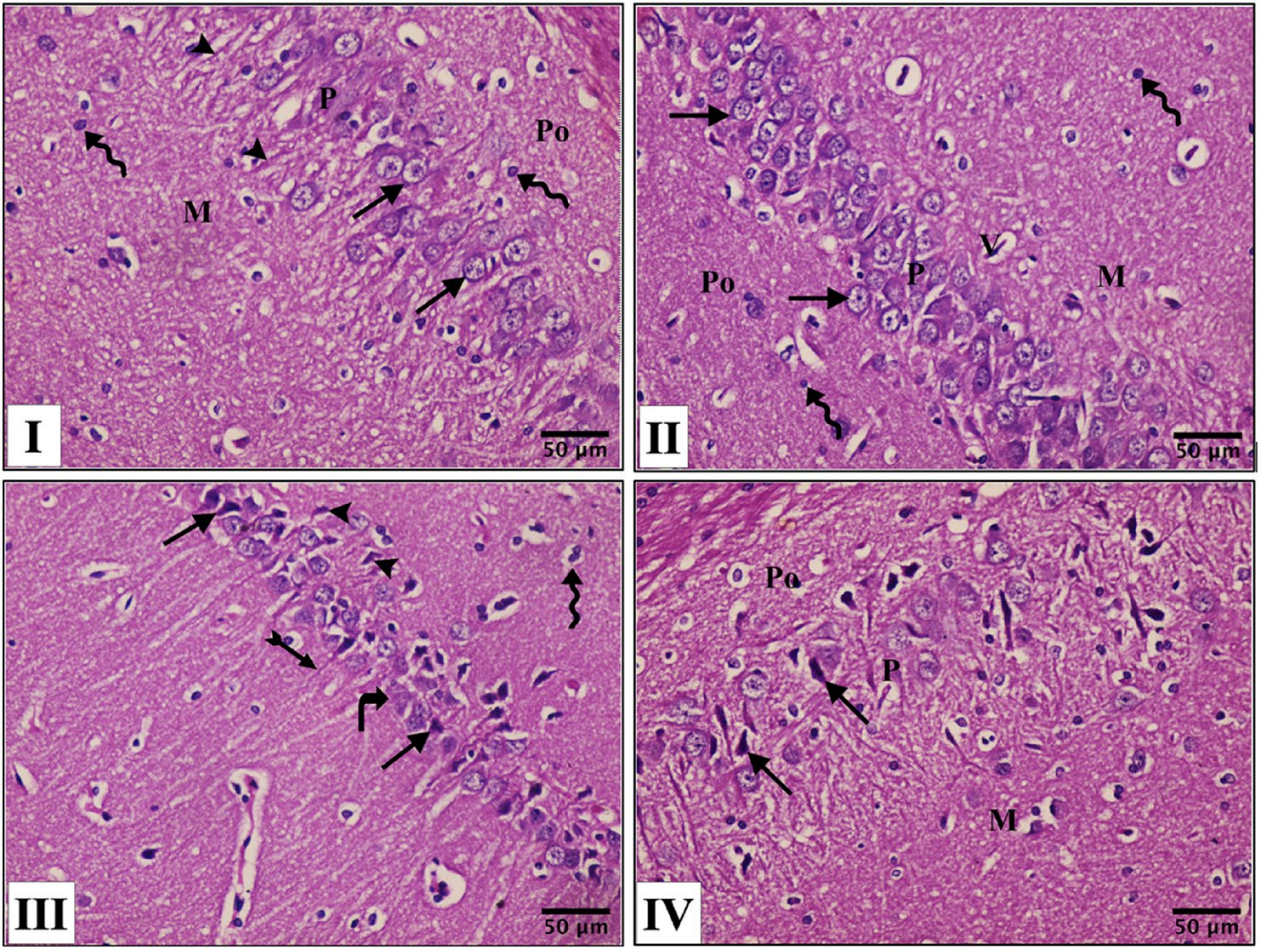
Effect of geraniol treatment on Hippocampal histopathological alternations in all the studied groups; **I** Control group, **II** geraniol group, **III** d-galactose group, **IV** D**-**galactose + geraniol group. Po: polymorphic layer; P: pyramidal layer; M: molecular layer; arrows: represent pyramidal cells with rounded nuclei with prominent nucleoli and basophilic cytoplasm in (I, II) but represent pyramidal cells with pyknotic nuclei and vacuolated acidophilic cytoplasm in (III, IV); arrow heads: apical dendrites extending to molecular layer in (I) but they represent pyramidal cells with fragmented nuclei in (III); wavy arrows: scattered neuroglial cells; V: scattered small blood vessels; curved arrows: represent pyramidal cells with ill-defined nuclei in (III); Tailed arrows: represent corkscrew dendrites of some pyramidal cells in the molecular layer in (III) (H&E. X 400, scale bar = 50 μm)

The hematoxylin and eosin-stained sections of the hippocampal CA1 area in all groups revealed that this area was composed of 3 layers; molecular, pyramidal, and polymorphic layers. In control and geraniol groups, the pyramidal layer is the main layer and it is composed of small pyramidal neurons containing rounded nuclei with prominent nucleoli and basophilic cytoplasm. Their apical dendrites were extended into the molecular layer (Fig. 4I, II). Chronic d-gal treatment (Group III) caused severely damaged neurons in CA1 area where many pyramidal cells have pyknotic, fragmented, and ill-defined nuclei. In addition, the cytoplasm of some cells was vacuolated and acidophilic. Also, the molecular layer exhibited corkscrew dendrites of some pyramidal cells, and the polymorphic layer exhibited that some neuroglial cells were fused together (Fig. 4III). The treatment with GE (Group IV) exhibited a neuroprotective response as most of pyramidal cells were apparently normal except for a few cells with pyknotic nuclei and cytoplasmic vacuoles (Fig. 4IV).

### The Effect of GE Treatment on Hippocampal Glial Fibrillary Acidic Protein (GFAP) Immunoreactivity in d-Gal Induced Aging Rats

**Fig. 5:**
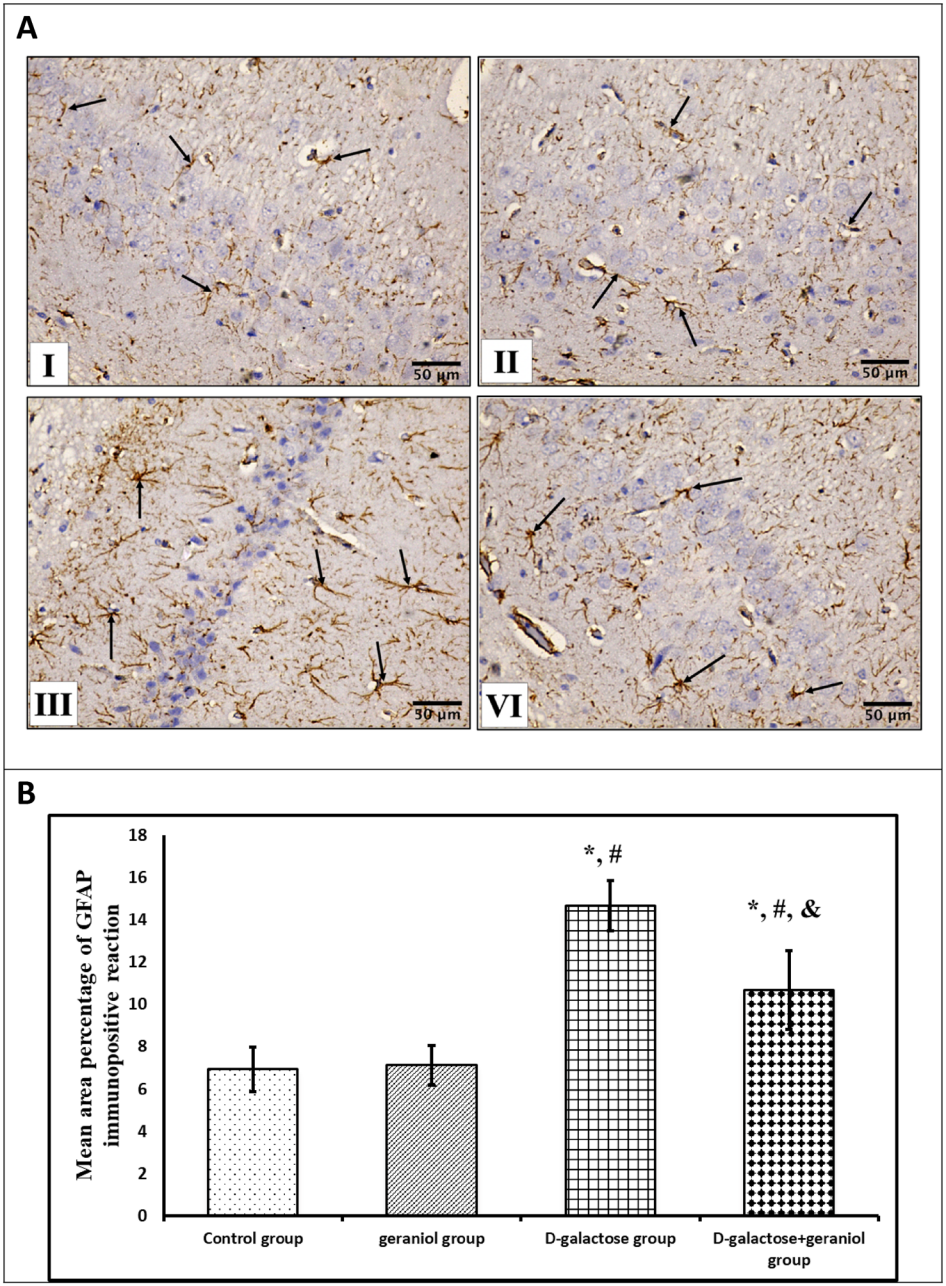
**A** Effect of geraniol treatment on hippocampal glial fibrillary acidic protein (GFAP) immunoreactivity (arrows) in all the studied groups; Control and geraniol groups (I & II) exhibit apparent few GFAP-positive reactive cells in CA1 area, d-galactose group (III) shows an apparent increased number of GFAP-positive reactive cells in CA1 area, and d-galactose + geraniol group (IV) shows an apparent decreased number of GFAP-positive reactive cells in CA1 area (GFAP X 400, scale bar = 50 μm). **B** Effect of geraniol treatment on the mean area percentage of GFAP immunopositivity reaction. Data were expressed as mean ± standard deviation. (n = 10/group). Statistical analysis was carried out using one-way ANOVA with Tukey's post hoc test, SPSS computer program. *Mean significant difference vs control group *(P* < *0.05) *^#^Mean significant difference vs geraniol group (*P* < 0.05). ^&^Mean significant difference vs d-galactose group (*P* < 0.05)

GFAP expression in the hippocampal CA1 area was examined in the present study to assess activated astrocytes. Immunostained sections from the control and geraniol groups (Group I and II) revealed few GFAP-positive reactive cells (Fig. 5AI, II). Meanwhile, d-gal group (Group III) revealed strong positive GFAP expression, indicating an increased number of activated astrocytes (Fig. 5AIII). Geraniol treatment (Group IV) ameliorated d-gal effects and showed an apparent decreased number of GFAP-positive reactive cells (Fig. 5AIV). The statistical analysis of morphometric results showed a significant increase in the area percentage of GFAP positive immunoreaction in group III (14.66 ± 1.2%) (P < 0.05) compared to groups I & II (6.94 ± 1.06, 7.12 ± 0.95% respectively), while group IV showed a significant decrease of the area percentage of GFAP positive immunoreaction (10.67 ± 1.85%) compared with group III (P < 0.05) (Fig. 5B).

### The Effect of GE Treatment on Hippocampal Caspase-3 Immunoreactivity in d-Gal-Induced Aging Rats

**Fig. 6:**
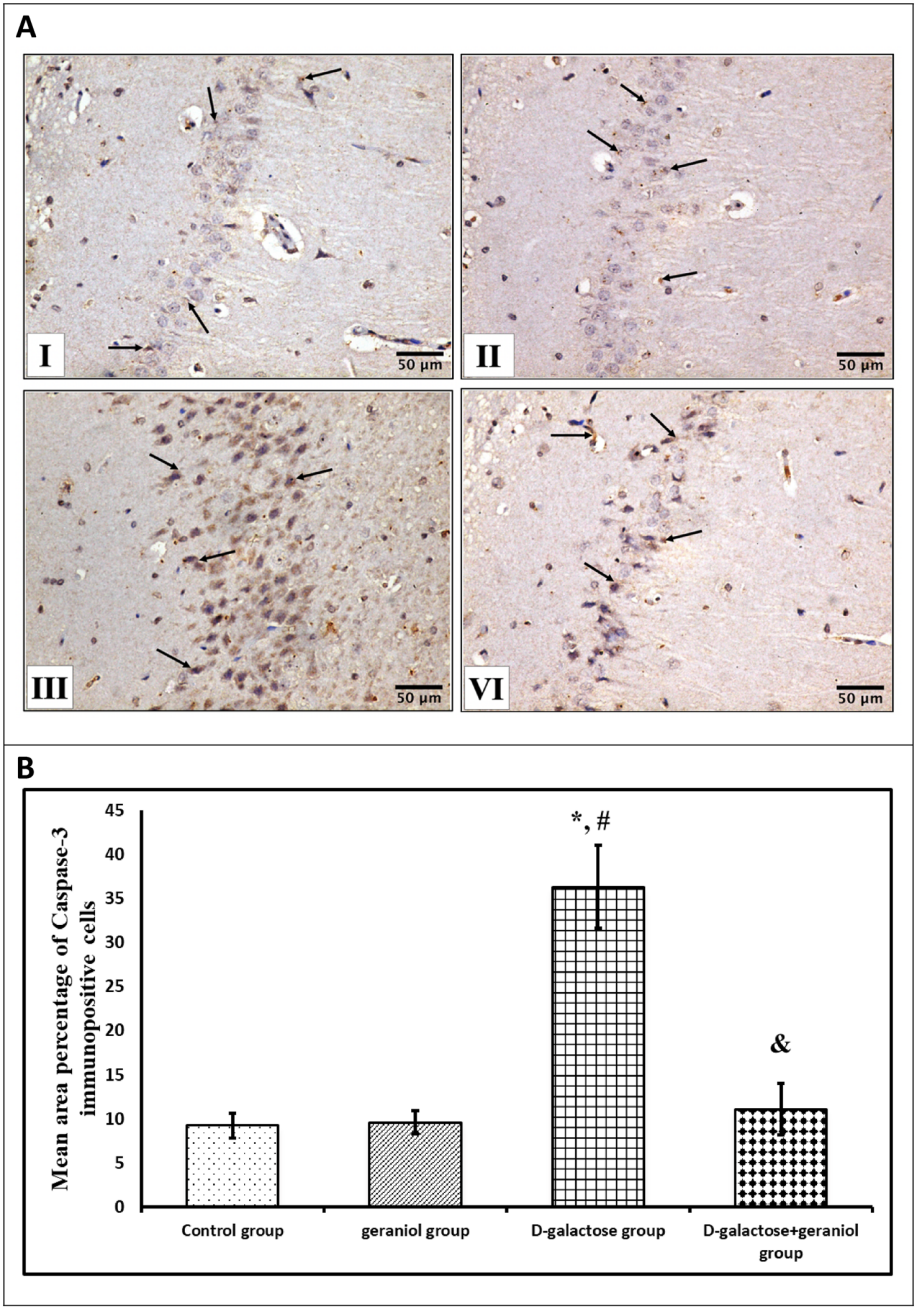
**A** Effect of geraniol treatment on hippocampal caspase-3 immunoreactivity (arrows) in all the studied groups; control and geraniol groups (I & II) exhibit apparent weak caspase positive reaction in the cytoplasm of pyramidal cells of CA1 area, d-galactose group (III) shows apparent strong caspase positive reaction, and d-galactose + geraniol group (IV) shows apparent moderate caspase positive reaction in the cytoplasm of pyramidal cells of CA1 area (Caspase-3 X 400, scale bar = 50 μm). **B** Effect of geraniol treatment on mean percentage of caspase-3 immunopositive cells in all the studied groups. Data were expressed as mean ± standard deviation. (n = 10/group). Statistical analysis was carried out using one-way ANOVA with Tukey's post hoc test, SPSS computer program. *Mean significant difference vs control group (*P* < 0.05) ^**#**^Mean significant difference vs geraniol group (*P* < 0.05). ^&^Mean significant difference vs d-galactose group (*P* < 0.05)

Immunostained sections from the control and geraniol groups (Group I and II) revealed faint immunoreaction for caspase-3 in the cytoplasm of pyramidal cells in the hippocampal CA1 area (Fig. 6AI, II). Meanwhile, the d-gal group (Group III) revealed a strong positive reaction in most of the hippocampal pyramidal cells (Fig. 6AIII). On the other hand, sections from group IV receiving d-galactose + geraniol exhibited apparent moderate caspase positive reaction in the cytoplasm of pyramidal cells of CA1 area (Fig. 6AIV). The statistical analysis of morphometric results showed a significant increase in the area percentage of caspase-3 positive immunoreaction in pyramidal cells of CA1 area in the d-galactose group (36.31 ± 4.73) (P < 0.05) compared to control and geraniol groups, while the pyramidal cells of CA1 area in d-galactose + geraniol group showed a significant decrease of the area percentage of caspase-3 positive immunoreaction (11.09 ± 2.91) (P < 0.05) compared to d-galactose group. On the other hand, galactose + geraniol group showed a non-significant difference in immunoreactivity compared to the control and geraniol groups (P > 0.05) (Fig. 6B).

## Discussion

Aging is an intrinsically complex process delineated by several changes at various levels of our biological hierarchy. The functional brain capability declines steadily as an intimate part of the aging process which is mirrored by a reduction in the cognitive performance including all domains; memory, learning, attention, and executive function [20].

Long-term treatment with d-galactose resulted in behavioral and neurochemical changes that mimic natural aging consequences in various tissues of preclinical animal models. Therefore, d-galactose is widely used for the pharmacodynamic evaluation of antidementia drugs [3].

In the current study, aging rat model induced by d-galactose was successfully established and applied to explore the potential role of geraniol in ameliorating d-gal induced memory impairment and understanding the possible mechanisms for its neuroprotective effect.

In the present study, the results of Morris water maze test showed obvious impairment of both short-term and long-term memory in d-galactose-administered rats. These results are in accordance with a previous study by Zhong et al. [8]. On the other hand, GE administration could efficiently improve the rats’ spatial memory; this was evidenced by the shortened escape latency along with the increased number of platform crossing. Furthermore, the results of the open field test indicated that GE significantly increased the exploratory ability in a novel environment. Collectively, these results indicated that GE exhibited a protective potential to d-gal-induced memory impairment.

These memory deficits may be confirmed by the histological alterations in area CA1 of the hippocampus of d-gal group where d-gal administration caused extensive damage in neurons in the hippocampus CA1 region. Additionally, many pyramidal cells have pyknotic fragmented and ill-defined nuclei and the cytoplasm of some cells was vacuolated and acidophilic. The results also revealed that GE treatment was significantly effective in reversing these changes.

Acetylcholine (Ach) has been considered as an important neurotransmitter involved in modifying learning, sleep, and memory. Enhanced cholinergic neurotransmission has been achieved by stimulating cholinergic receptors as well as increasing the availability of Ach in neuronal synaptic cleft [21]. AchE is a key cholinergic enzyme regulating the level of Ach in the brain [22] and the changes in its activity are strongly correlated with learning decline and memory deficits [23].

Our study displayed a significant increase in AchE activity in rats following the chronic administration of d-gal. This increase may be attributed to genetic overexpression of this enzyme induced by d-gal-associated oxidative stress [24]. Consistent with these findings, d-gal significantly decreased cognitive functions as a consequence of increased AchE activity. In contrast, our results showed that GE markedly reversed changes in the AchE activity and Ach level. These results are in accordance with the results of Prasad [12]. Accumulating evidence revealed that the brain has the highest oxygen demand in the body and weak antioxidant mechanism. Therefore, it is highly susceptible to oxidative damage than other organs [25]. d-gal in high concentration can be metabolized into aldose and hydrogen peroxide by galactose oxidase, resulting in the generation of ROS. Normally, there is an intimate balance between the rate of ROS generation and their neutralization by the antioxidant defense system. However, the excessive generation of ROS and the decrease in antioxidant enzymes’ activity in the brain result in tissue damage [4].

To clarify this idea, MDA, a lipid peroxidation marker, SOD, and reduced glutathione were estimated. Noteworthy, our results revealed that systemic chronic administration of d-gal induces oxidative stress; this is evidenced by decreased SOD activity and GSH content as well as increased MDA level. These findings are in accordance with a previous study suggesting that d-gal enhanced ROS generation has been associated with disruption of oxidant/antioxidant balance [26].

Meanwhile, as shown in the current study, redox equilibrium in rats was recovered significantly following GE treatment, suggesting that GE can protect the brain from oxidative damage in the progress of aging. This anti-oxidant effect of GE detected in our results is in accordance with previous works by Prasad and Wang et al. [12, 27].

Concomitantly, ROS can trigger inflammatory responses via the activation of NF-kβ, a redox-sensitive transcription factor, inducing the expression of various inflammatory mediators such as TNF-α, IL-6, and IL-1β. Moreover, sustained inflammation resulted in a likely vicious cycle of progressive ROS generation [26]. This notion may explain partially the d-gal-induced inflammatory response and oxidative stress observed in our study. In previous studies, GE showed its anti-inflammatory effects via decreasing inflammatory mediators both in vivo and in vitro [28]*.* This is supported by our finding that d-galactose + geraniol group showed a significant decrease in the levels of TNF-α, IL-1β, and IL-6 compared to d-galactose group.

Moreover, NF-kβ activation was stimulated by numerous cellular kinases involving MAPKs which constitute a highly conserved family of protein serine/threonine kinases involving ERK, P38, and JNK subfamilies [29]. In the current research, chronic d-galactose administration resulted in increased phosphorylated levels of p38MAPK and JNK. These results suggest that MAPK pathway is an effective target for anti-inflammatory and anti-neurodegenerative drugs.

The administration of GE plus d-gal caused a significant decrease in the phosphorylated level of p38MAPK and JNK compared to d-galactose group. These results suggest that GE decreased inflammatory mediator production via the inhibition of MAPK signaling pathway. This anti-inflammatory effect of GE is in line with some previous studies [27, 30]. The results of these previous studies suggested that geraniol's neuroprotective effects in d-gal memory impairment could be mediated through modulating NF-kβ/MAPK signaling pathway.

Astrocytes play several roles in supporting neurons and viable synaptic transmission required for memory consolidation. Thus, GFAP is used as an important marker for activated astrocytes in several models [31]. In our experiment, d-gal treatment provoked a significant increase in GFAP expression in rat's hippocampus, suggesting astrocytes activation. The latter may be attributed to the activated NF-kβ induced by oxidative stress [21]. In parallel with these results, age-dependent activation of astrocytes and increased GFAP level were previously reported in the study of Santello et al. [31]. In addition, GE plus d-gal administration in the current study decreased GFAP expression in the rats’ brains, suggesting the neuroprotective effect of geraniol. This finding is supported by a previous research by Lv et al. [32].

BDNF, an important neurotrophic growth factor, is highly expressed in the rat’s brain tissue [33]. It is considered as an important factor in the control of numerous neurocognitive functions such as synaptic transmission, plasticity, learning, and memory. Furthermore, BDNF activates the NF-kB signaling cascade to regulate inflammatory substances such as inflammatory cytokines. Therefore, BDNF is an important mediator between cognitive impairment and neuroinflammation. The current work revealed that chronic d-gal administration significantly decreased BDNF level that was significantly reversed upon GE treatment. These results agree with earlier results of Jiao et al. and Rekha et al. [33, 34].

Surprisingly, compelling evidence suggested that oxidative stress and inflammation are 2 mechanisms working in harmony to induce apoptosis in d-gal induced tissue injury. Caspase-3, one of the major apoptotic mediators, may be activated by elevated ROS, causing neuronal dysfunction [35]. In line with these findings, our study revealed that caspase-3 activity was elevated in the hippocampus of d-gal treated rats, while GE treatment of d-gal-treated rats resulted in the restoration of elevated caspase-3 activity. These results suggest that geraniol's anti-apoptotic effect may be due to its ability to regulate the expression of apoptosis-related proteins through ameliorating oxidative stress. Our results are in line with the results of a previous study by Vinothkumar et al. [36].

ER stress is tightly linked with the accumulation of unfolded/misfolded proteins evoking UPR signaling [5]. The latter is mediated basically by dissociating glucose-regulated protein (GRP78) chaperone from its three ER receptors (IRE1, PERK, and ATF6) [37].

Aging is associated with decreased cells’ ability to cope with protein folding, accumulation, and aggregation. This is associated with the failure of key ER molecular chaperones and folding enzymes, thus compromising the UPR [38]. The initiation of ER stress-related apoptotic signaling was mediated in response to accumulated misfolded proteins by auto-phosphorylation of the IRE1α kinase domain. The latter activates the RNase to induce the splicing of X-box binding protein 1 (XBP1), a critical ER stress sensor, which triggers pro-apoptotic signaling pathway [38]. Moreover, PERK phosphorylates the eukaryotic initiation factor eIF2α, conducting apoptosis by activating CHOP which is the main determinant of cell fate and ER stress-induced apoptosis [39].

In line with the above-mentioned data, d-gal treated rats in the current study showed increased GRP78 expression with subsequent increase in PERK and IRE-1α levels, resulting in ER stress-mediated apoptosis that was linked with the up-regulation of CHOP expression and elevated caspase-3 activity in rats' hippocampus.

Notably, GE administration significantly offsets ER-stress-induced apoptosis in d-gal aged rats as confirmed by GRP78 down-regulation with concomitant decrease of PERK and IRE-1α levels. Additionally, a considerable down-regulation of CHOP expression and lowered caspase-3 activity were detected after GE treatment. Previous studies supported the protective effects of GE in metabolic disorders and Parkinson’s disease, through the down-regulation of ER stress markers and amelioration of ER-stress-induced apoptosis [40].

## Conclusion

In summary, our findings revealed that GE could recover d-gal memory deficits through attenuating oxidative damage, inflammation, and hippocampal neuronal damage. The effect of GE may be mediated through the inhibition of NF-kβ/MAPK signaling pathway and regulating ER stress-mediated apoptotic pathways. Moreover, GE has a cytoprotective effect on hippocampal neurons and could reverse abnormalities in the cholinergic system function and decrease GFAP expression in rat's brain. These findings suggest that GE may be a potential candidate for further preclinical studies on the treatment of aging- related memory deficits.

## Data Availability

Data will be made available on reasonable request.

## Abbreviations

ANOVA: One-way analysis of variance
Ach: Acetyl Choline
AchE: Acetylcholinesterase enzyme
ATF6: Activating transcription factor 6
BDNF: Brain-derived neurotrophic factor
CHOP: C/EBP homologous protein
d-Gal: d-Galactose
eIF2α: Eukaryotic initiation factor 2 alpha
EL: Escape latency
ER: Endoplasmic reticulum
ER UPR: Endoplasmic stress unfolding protein response
GE: Geraniol
GFAP: Glial fibrillary acidic protein
GSH: Reduced glutathione
GRP78: Glucose-regulated protein 78
H&E: Hematoxylin and Eosin
i.p: Intraperitoneal
IL-1β: Interleukin 1 beta
IL-6: Interleukin-6
IRE1: Inositol requiring enzyme-1
JNK: C-Jun N-terminal kinase
MAPKs: Mitogen-activated protein kinases
MDA: Malondialdehyde
MWM test: Morris water maze test
NF-kB: Nuclear factor kappa beta
PBS: Phosphate-buffered saline
PERK: Protein kinase RNA-like endoplasmic reticulum kinase
ROS: Reactive oxygen species
SCI: Spinal cord injury
SOD: Superoxide dismutase
TBA: Thiobarbituric acid
TNF-α: Tumor necrosis factor-alpha
